# Tight Junction Proteins at the Crossroads of Inflammation, Barrier Function, and Disease Modulation

**DOI:** 10.3390/ijms26178115

**Published:** 2025-08-22

**Authors:** Hee-Jae Cha

**Affiliations:** Department of Parasitology and Genetics, Kosin University College of Medicine, Busan 49267, Republic of Korea; hcha@kosin.ac.kr

## 1. Tight Junctions: From Static Barriers to Dynamic Regulators

Tight junctions (TJs), traditionally considered static structural elements sealing epithelial and endothelial monolayers, are now recognized as dynamic regulators of tissue homeostasis [1,2]. They play pivotal roles in immune modulation, microbial interaction, nutrient sensing, and signal transduction [3,4,5]. Recent studies have highlighted the involvement of TJ proteins in various pathological conditions, including inflammatory disorders, metabolic dysregulation, barrier dysfunction, and cancer metastasis [6,7,8,9]. The five original research articles in this Special Issue of the *International Journal of Molecular Sciences* exemplify this paradigm shift, offering new insights into the multifaceted roles of TJs (Table 1).

## 2. Natural Agents Restore Barrier Function in Inflammatory Bowel Disease

Ulcerative colitis (UC), a subtype of inflammatory bowel disease (IBD), is characterized by chronic inflammation and compromised intestinal barrier function. Two studies in this Special Issue explore natural therapeutics that confer protective effects via TJ regulation. In a murine model of DSS-induced colitis, the administration of Candida utilis (CUM) was found to significantly mitigate disease severity by restoring the expression of TJ proteins (Claudin-3, Occludin, and ZO-1), suppressing inflammatory cytokines (IL-1β, IL-6, TNF-α), and modulating microbial communities by increasing the abundance of beneficial bacteria such as Lactobacillus and Prevotellaceae. Moreover, CUM preserved short-chain fatty acid (SCFA) production and downregulated NF-κB, MAPK, and PPARγ pathways, suggesting a multifactorial mechanism of gut protection. Similarly, Polycan, a β-glucan polysaccharide purified from Aureobasidium pullulans, displayed potent anti-colitic properties. Both 250 mg/kg and 500 mg/kg dosages prevented body weight loss, colon shortening, and histological damage in DSS-treated mice. Polycan significantly preserved TJ function, decreased permeability, and reversed dysbiosis, showing comparable efficacy to 5-aminosalicylic acid (5-ASA). These findings highlight the therapeutic value of natural immunomodulators that enhance barrier resilience through TJ preservation.

## 3. Dietary Fat and Ketone Bodies: Friend or Foe to the Intestinal Barrier?

The metabolic regulation of TJ integrity is an emerging area of interest. A clinical study included in this issue investigates how a high-fat diet (HFD), which induces intestinal ketogenesis via PPARα and HMGCS pathways, impacts intestinal permeability and TJ protein expression in healthy adults. While PPARα signaling was altered in response to the ketogenic shift, and in vitro studies indicated subtle changes in TJ regulation (e.g., Claudin-3 upregulation with ketone inhibition), the in vivo human data revealed no significant change in barrier permeability or TJ expression following short-term HFD intervention. These results suggest that while intestinal ketogenesis may influence TJ dynamics under certain conditions, the healthy human gut may compensate for macronutrient-induced stress without manifesting overt barrier dysfunction—at least in the short term.

## 4. Occludin: A Rediscovered Gatekeeper in Pulmonary Inflammation

Traditionally deemed dispensable in some tissues, occludin is re-evaluated in the context of acute respiratory distress syndrome (ARDS), a life-threatening pulmonary condition marked by alveolar barrier disruption. Through both patient tissue analysis and murine gene delivery models, the study demonstrated that occludin downregulation correlates with lung injury, while its overexpression dampens endotoxin-induced damage. Loss of occludin in mice led to impaired barrier integrity, elevated protein content in bronchoalveolar lavage fluid, and histological lung injury. Furthermore, occludin expression positively correlated with claudin-18 localization in alveolar epithelial cells. This study repositions occludin as an essential barrier regulator in pulmonary inflammation and opens avenues for occludin-targeted therapeutics in ARDS.

## 5. Tight Junction Scaffolds as Tumor Suppressors in Melanoma

The oncogenic consequences of TJ protein loss are exemplified in a study utilizing CRISPR–Cas9-mediated knockout of Tjp1 and Tjp2 (ZO-1 and ZO-2) in B16-F10 melanoma cells. Knockout cells exhibited significantly increased proliferation, migration, invasion, and metastatic capacity both in vitro and in vivo. Transcriptomic analyses revealed the dysregulation of genes related to cell cycle progression, angiogenesis, and adhesion, including a notable decrease in Ninjurin-1 and Catenin alpha-1. Claudin-1 expression was also reduced in Tjp-KO cells, reinforcing the concept that TJ integrity restrains malignant transformation. These findings align with the growing evidence that TJ scaffold proteins function as tumor suppressors by maintaining epithelial architecture and suppressing migratory phenotypes.

## 6. Conclusions

Taken together, the studies featured in this Special Issue offer a multidimensional view of tight junction proteins—as mediators of immune regulation, metabolic balance, microbial interaction, and cancer suppression. Far from passive structural components, TJ proteins have emerged as molecular sentinels of epithelial resilience, dynamically responding to environmental, microbial, and systemic cues. By elucidating the interplay between TJ proteins and various pathological stimuli, these investigations move the field toward translational applications. Whether through probiotic supplementation, metabolic modulation, or gene-based therapy, targeting TJs represents a promising avenue for therapeutic development in inflammatory, infectious, and neoplastic diseases. As the field advances, further exploration of TJ signaling pathways may unveil novel strategies to fortify barrier function and combat disease at its origin—the cell junction.

## Figures and Tables

**Table 1:** Summary of original research on tight junction functions and disease.

Study	Model/System	Main Focus	Key Findings	TJ Proteins Involved
*Candida utilis* in UC	DSS-induced colitis (mice)	Anti-inflammatory effects	Inflammation ↓, SCFAs, gut microbiota modulation ↑	Claudin-3, Occludin, ZO-1
Polycan in UC	DSS-induced colitis (mice)	Natural β-glucan protection	Permeability ↓, apoptosis ↓, microbiota diversity ↑	Not specified, TJ integrity restored
HFD and ketogenesis	Human volunteers + in vitro	Diet-induced permeability	PPARα modulation, no permeability change in vivo	Claudin-3 (in vitro ↑), others unaffected
Occludin in ARDS	ARDS patients + mice	Lung barrier integrity	Occludin ↓ = injury ↑, gene therapy rescued damage	Occludin, Claudin-18
Tjp1/Tjp2 KO in melanoma	B16-F10 melanoma (in vitro/in vivo)	Tumor progression	Proliferation ↑, Claudin-1 ↓, adhesion genes ↓	Tjp1, Tjp2, Claudin-1
